# A narrative review of wastewater surveillance: pathogens of concern, applications, detection methods, and challenges

**DOI:** 10.3389/fpubh.2024.1445961

**Published:** 2024-07-30

**Authors:** Surabhi Singh, Amina Ismail Ahmed, Sumayya Almansoori, Shaikha Alameri, Ashraf Adlan, Giovanni Odivilas, Marie Anne Chattaway, Samara Bin Salem, Grzegorz Brudecki, Wael Elamin

**Affiliations:** ^1^Microbiology Lab, Reference and Surveillance Intelligence Department, Abu Dhabi, United Arab Emirates; ^2^United Kingdom Health Security Agency, Gastrointestinal Bacteria Reference Laboratory, London, United Kingdom; ^3^Central Testing Laboratory, Abu Dhabi Quality and Conformity Council, Abu Dhabi, United Arab Emirates

**Keywords:** wastewater surveillance, infectious disease, pathogens, detection methods, challenges, public health, epidemiology

## Abstract

**Introduction:**

The emergence and resurgence of pathogens have led to significant global health challenges. Wastewater surveillance has historically been used to track water-borne or fecal-orally transmitted pathogens, providing a sensitive means of monitoring pathogens within a community. This technique offers a comprehensive, real-time, and cost-effective approach to disease surveillance, especially for diseases that are difficult to monitor through individual clinical screenings.

**Methods:**

This narrative review examines the current state of knowledge on wastewater surveillance, emphasizing important findings and techniques used to detect potential pathogens from wastewater. It includes a review of literature on the detection methods, the pathogens of concern, and the challenges faced in the surveillance process.

**Results:**

Wastewater surveillance has proven to be a powerful tool for early warning and timely intervention of infectious diseases. It can detect pathogens shed by asymptomatic and pre-symptomatic individuals, providing an accurate population-level view of disease transmission. The review highlights the applications of wastewater surveillance in tracking key pathogens of concern, such as gastrointestinal pathogens, respiratory pathogens, and viruses like SARS-CoV-2.

**Discussion:**

The review discusses the benefits of wastewater surveillance in public health, particularly its role in enhancing existing systems for infectious disease surveillance. It also addresses the challenges faced, such as the need for improved detection methods and the management of antimicrobial resistance. The potential for wastewater surveillance to inform public health mitigation strategies and outbreak response protocols is emphasized.

**Conclusion:**

Wastewater surveillance is a valuable tool in the fight against infectious diseases. It offers a unique perspective on the spread and evolution of pathogens, aiding in the prevention and control of disease epidemics. This review underscores the importance of continued research and development in this field to overcome current challenges and maximize the potential of wastewater surveillance in public health.

## 1 Introduction

Recent decades have seen a rise in both the emergence and reemergence of pathogens, which has led to significant and deadly outbreaks (1, 2, 3). Authorities such as the global scientific community, the National Institutes of Health (NIH), USAID, and the World Health Organization (WHO) are aware of the substantial worldwide impact these outbreaks have and the importance of developing predictive and preventive systems. Since 1970, there has been the identification of over 1,500 new pathogens, with about 40 being deemed emerging infectious diseases (4). Regular mass screening in clinical settings poses difficulties, and those who are asymptomatic or exhibit mild symptoms frequently go undetected. The increase in the global population is likely to escalate these challenges and the risk of infectious diseases, highlighting the need for a surveillance method that is comprehensive, provides real-time results, can monitor multiple diseases—including rare ones—and is both scalable and cost-effective. Wastewater surveillance historically serves to monitor water-borne or fecal-orally transmitted pathogens by collecting samples from sewage systems, offering a sensitive way to observe changes and varieties of pathogens within communities (5). Over the past three decades, studies have consistently shown the accuracy of wastewater testing in representing disease at the population level (6). Chemical and biological markers in wastewater could even act as an early alert system for disease breakouts, potentially improving current surveillance systems for infections (7). The origins of wastewater surveillance can be traced to John Snow’s seminal work on London’s cholera outbreak in 1854, where he identified contaminated water as a primary source (8–10). In the 1940s in the United States, wastewater was pivotal for tracking and managing polio outbreaks, with poliovirus detection still considered highly sensitive today, becoming common practice in many parts of the world (11, 12).

The advantage of sampling wastewater lies in its high pathogen content compared to other environmental samples (13, 14). It also allows for the inclusion of pathogens from individuals who are either asymptomatic or pre-symptomatic, unlike clinical tests, thus presenting a potent early indicator and prompt intervention tool for infectious diseases. Moreover, recent interest has emerged in using wastewater examination for AMR (antimicrobial resistance) surveillance, with studies revealing seasonal distributions of AMR, worldwide gene abundance, and correlations between AMR found in wastewater and clinical contexts (15, 16, 17).

Despite various reviews discussing wastewater surveillance’s significance, there’s a gap in literature providing a thorough review that collectively highlights concerning pathogens, wastewater surveillance applications, available technologies, and pathogen detection challenges in wastewater. Thus, this narrative review focuses on wastewater surveillance for infectious diseases, aiming to consolidate these issues. In preparing this narrative review, a methodical approach was used, using a selection of prominent medical search engines to ensure a comprehensive exploration of the literature. The databases harnessed for this review included PubMed, Scopus, ScienceDirect, The Cochrane Library, and Google Scholar. Only published studies were included for this review. Non–peer-reviewed articles such as short communications and research letters were excluded.

The methodology entailed a systematic and structured search using a set of predetermined search terms that were central to the theme of wastewater surveillance and its role in public health. These terms included “wastewater surveillance,” “pathogens,” “detection methods,” “public health,” and “epidemiology,” among others. The search was refined to capture articles that shed light on the methodologies for pathogen detection in wastewater, the challenges encountered in the surveillance process, and the implications for public health policy and disease prevention.

## 2 Wastewater surveillance: monitoring key pathogens of concern

**Figure 1:**
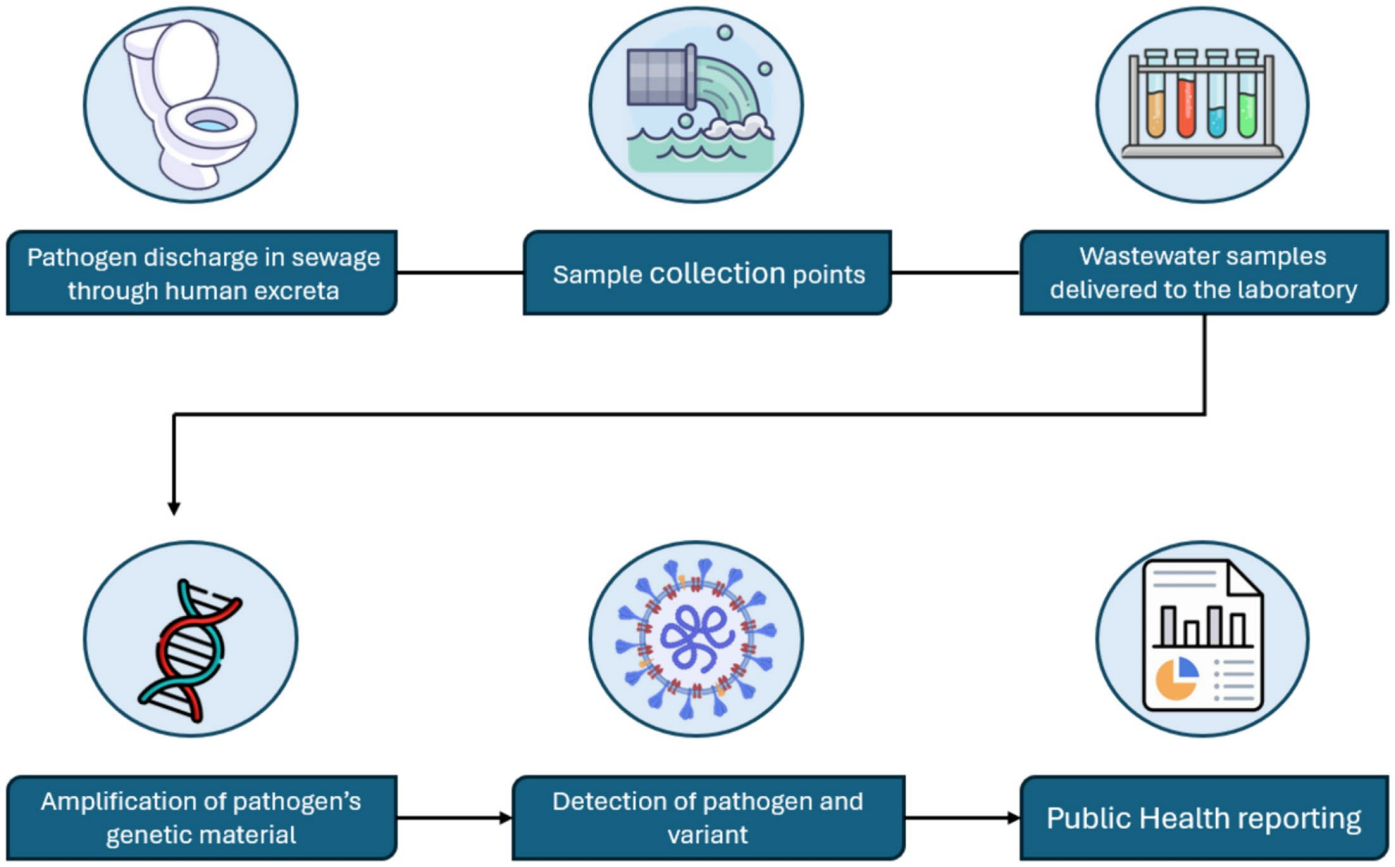
Wastewater surveillance pathway.

Human pathogens, causing infections and even death, remain a leading threat to global public health. Currently, there are approximately 538 species of pathogenic bacteria, 208 viruses, 57 species of parasitic protozoa and some fungi and helminths infecting humans (24, 25). Numerous pathogen species found in wastewater pose a serious threat to human health. Different type of pathogens and concerned diseases have been listed in Table 1. Also, the pathway for and effective wastewater surveillance has been explained in Figure 1.

**Table 1:** Major pathogens of concern in water system and relatable diseases.

Pathogens	Associated disease	Concentration in wastewater	*Health impact
Bacteria			
*Campylobacter* spp.	Diarrhea, gastroenteritis	Medium to high	High
*Yersinia enterocolitica*	Diarrhea, reactive arthritis		High
*Escherichia coli*	Acute diarrhea, bloody diarrhea and gastroenteritis		High
*Burkholderia pseudomallei*	Melioidosis		High
*Salmonella typhi*	Typhoid fever, paratyphoid fever and other serious salmonellosis		High
*Shigella* spp.	Bacillary dysentery or shigellosis		High
*Vibrio cholerae*	Cholera, gastroenteritis		High
Virus			
Adenovirus	Gastroenteritis	Medium to high	High
Enterovirus	Gastroenteritis		High
Hepatitis A virus	Hepatitis		High
Hepatitis E virus	Infectious hepatitis; miscarriage and death		High
Rotavirus	Gastroenteritis		High
Sapovirus	Gastroenteritis		High
Norovirus	Gastroenteritis		High
Protozoa			
*Cryptosporidium cayetanensis*	Diarrhea	Low to medium	High
*Giardia intestinalis*	Diarrhea		High
*Entamoeba histolytica*	Acute amoebic dysentery		High
*Giardia duodenalis*	Giardiasis		High

Data obtained from Zhang et al. (18), Ramirez et al. (19), Fijalkowski et al. (20), Ahmed et al. (21), EPA (22), and WHO (23).*Health significance relates to the severity of impact, including association with outbreaks.

Most pathogens in wastewater are shed by humans, although some might originate from other sources such as animals. Some of these pathogens have been discussed in detail below.

### 2.1 Gastrointestinal pathogens

*Campylobacter* spp. is major cause of diarrhea, and human gastroenteritis worldwide (48). It is comprised of 17 species and 6 subspecies, out of which *Campylobacter jejuni* and *Campylobacter coli* account for 80–85% and 10–15% of total infections, respectively (Leblanc et al., 2011) and are also the main species widely detected and isolated from wastewater (49, 50). *C. jejuni* was first isolated from the feces of patients with gastrointestinal disease in the 1970s (51). Subsequently, many studies have demonstrated *C. jejuni* to be a major cause of human infections (52) transmitted by the fecal-oral route through contaminated food and water (53).

*Salmonella* is another important enteropathogenic bacteria, causing approximately 94 million infections and 155,000 deaths annually worldwide (54, 55). *Salmonella enterica* serovar Typhi and *Salmonella enterica* serovar Paratyphi are the main causes of typhoid fever and paratyphoid fever, respectively (56, 57). Both are gram-negative, human-restricted, and species-specific bacterial diseases. The transmission can occur from person to person by eating contaminated food or water or by contact with an acute or chronic infected person (58, 59). To evaluate the water quality and the likelihood of contracting waterborne infections, a study was carried out in Nigeria that examined several sources of drinking water (19). Areas with a high number of reported waterborne cases and those with a low number of cases had their water samples taken. Most tests contained *Vibrio cholerae*, *Salmonella typhi*, and *Shigella dysenteriae*, and it was hypothesized that discharge of polluted water during the intense rainy season had contaminated drinking water sources (19).

Enterohaemorrhagic and enteroinvasive *Escherichia coli* are pathogenic and causes illness in mammals including humans. Shiga toxin producing *E. coli* (STEC) O157:H7 causes diarrhea, haemorrhagic colitis, haemolytic uremic syndrome, that leads to serious long-term complication, and it is often employed as a model for pathogenic bacteria study in wastewater (20). Through PCR, high amount of *E. coli* O157:H7 gene were detected in the sewage sludge (1,819,700 copies of gene/100 mL). The common feature of STEC *E. coli* O157:H7 is that even a low inoculum as little as 10 cells may trigger disease (60). In 2000, an outbreak in Walkerton, Ontario was linked to *E. coli* O157:H7 in the Great Lakes area, resulting in 2300 illness cases (61). In 2011 in Germany, a STEC *E. coli* (strain O104:H4) was the causative agent of severe cases of acute diarrhea and bloody diarrhea due to the consumption of uncooked sprouts that were irrigated with contaminated water (62).

The protozoan parasites, Cryptosporidium and Giardia, are also important enteric pathogens of public health concern and major waterborne pathogens (63, 64). Cryptosporidium is the second most important cause of moderate to severe diarrhea and mortality in children under 5 years of age in developing countries (65). The largest cryptosporidiosis outbreak due to Cryptosporidium protozoa occurred in 1993 in United States, which affected over 400,000 individuals, was due to drinking water becoming contaminated with wastewater (66). Giardiasis is the most common enteric protozoan parasitic infection worldwide, with an estimated 280 million people infected annually (67). Both parasites are prevalent in wastewater with concentrations in as high as 60,000 Cryptosporidium oocysts and 100,000 Giardia cysts (68).

Among viruses, Adenoviruses are a leading pathogen of clinical diseases, such as gastroenteritis, conjunctivitis, respiratory illnesses, haemorrhagic cystitis, and systemic infections. Adenoviral infections accounts for 2 to 10% cases of diarrhea. They are commonly detected in raw wastewater and have been cited as among the most significantly abundant human viruses in wastewater. Adenoviruses have also been detected in human excrement of infected persons, including both feces and urine (69).

In both low to middle-income and high-income countries, Norovirus is considered the second main cause of viral acute gastroenteritis after rotavirus. Globally, norovirus is responsible for nearly 20% of all acute gastroenteritis cases, with 677 million cases per year and over 213,000 deaths. Studies have linked the level of enteric viruses such as Norovirus, Hepatitis E and Hepatitis A virus in wastewater with incidence of clinical cases. Hence, wastewater surveillance can provide an early warning of outbreaks involving enteric viruses (70, 71).

### 2.2 Respiratory pathogen

The emergence in 2020 of the severe acute respiratory syndrome Coronavirus 2 (SARS-CoV-2), which causes viral pneumonia, has heightened the focus on Wastewater as a surveillance tool to provide early detection of disease in the community. There are more than 2,000 locales in 55 nations where wastewater surveillance for SARS-CoV-2 is ongoing, and there are many cases across the literature reporting on the detection of SARS-CoV-2 from sewage (72). Although SARS-CoV-2 typically causes respiratory symptoms, and is shed in nasal, buccal, esophageal, and respiratory discharges into wastewater, it can also result in gastrointestinal symptoms and/or viral shedding in feces (73, 74). In a meta-analysis of COVID-19 studies, finding revealed that 17.6% of COVID-19 patients had gastrointestinal symptoms and 48.1% of COVID-19 patients had SARS-CoV-2 RNA detected in their feces. Thus, monitoring the presence of SARS-CoV-2 RNA in wastewater is becoming widely used to track changes in COVID-19 case numbers in communities.

Among other respiratory pathogens, 13 respiratory viruses were detected from different wastewater treatment plants in Queensland, Australia. Out of these 13 viruses, Bocavirus (BoV), Parechovirus (PeV), Rhinovirus A (RhV A) and Rhinovirus B (RhV B) were detected in all wastewater samples (21). Different studies reported here shows that the application of wastewater surveillance to monitor respiratory viruses can be a potential tool in community disease surveillance.

## 3 Application of wastewater surveillance

### 3.1 Understanding outbreaks and public health through wastewater studies

The detection of the Polio virus nationwide in late 1930s United States sewers (75), the presence of non-polio enteroviruses in the Philippines’ children (76), and recent traces in New York (77, 78) and London (79, 80) highlighted the need for swift governmental action against potential outbreaks.

Detection of SARS-CoV-2, Mpox virus and PMMoV in community wastewater of United States was evaluated by Keegan et al. (81). A study done in Hong Kong Zheng reported that wastewater surveillance can even provide spatiotemporal SARS-CoV-2 infection dynamics (82). Wolken et al. (83), in Houston demonstrated role of wastewater surveillance in detection of SARS CoV-2 and Influenza outbreaks. Similarly, Evidence of SARS-CoV-2 in Australian wastewater was presented by Ahmed et al. (84), shedding light on community prevalence and aiding public health measures (85, 86). Hasan et al. (87), and Vo et al. (88) completed further wastewater studies in the UAE, discovering early indications of SARS-CoV-2 variants prior to clinical case identification. Kirby et al. (89) detected omicron mutation markers in the United States sewage, underscoring the predictive capability of wastewater-based epidemiology.

In South Africa, a study done by Yousif et al. (90), demonstrated the utility of wastewater genomics to monitor evolution and spread of endemic viruses. Investigation in Sweden by Hellmér et al. (91), using qPCR found substantial amounts of Norovirus GII and Hepatitis A indicating upcoming outbreaks. This technique allows estimation of affected individuals based on viral load in sewage. Countries like Spain and United States with documented clinical cases and community spread detected the Mpox virus in wastewater samples (92, 93). In Nepal, *Salmonella typhi* bacteriophages were detected from surface waters which was reported as a scalable approach to environmental surveillance (94).

Rechenburg and Kistemann (95) found Campylobacter contamination in German rivers increased infection risks, while Liu et al. (58), reported typhoid-causing bacteria in India and Bangladesh’s wastewater. Diemart and Yan’s study (96) exposed undiscovered *S. enterica* outbreaks linked to wastewater strains via genetic analysis. Barrett et al. (97), isolated *Vibrio cholerae* O1 from Louisiana sewage, and Zohra et al. (98), identified toxigenic strains in Pakistan’s water presenting continual infection threats unrelated to season patterns.

Razzolini et al. (99), disclosed a high frequency of Cryptosporidium and Giardia in Brazilian chlorine-treated wastewater, leading to gastrointestinal disease transmission through poor hygiene. Additionally, Amoah et al. (100), observed multiple parasites in South African wastewater, with particular concern for worm-infested community water sources as evidenced by a Monte Carlo study (101).

These comprehensive wastewater surveillance studies aid in formulating public health policies and establishing outbreak response, demonstrating their value in epidemiological research.

### 3.2 Antimicrobial resistance detection in wastewater

One of the major factors affecting the re-emergence of infectious diseases is antimicrobial resistance (102). According to the United Nations, around 700,000 people die yearly of infections associated with antimicrobial resistant microorganisms. Wastewater is one of the primary routes for resistant pathogens and antimicrobe to enter the environment.

Mao et al. (103) studied prevalence of antibiotic resistance genes reported in wastewater treatment plants. Similarly (104), studied diverse range of multiple antibiotic resistance genes in 10 large-scale membrane bioreactors for municipal wastewater treatment. The effects of seasonality upon antibiotic resistance genes in wastewater is another underexplored area, though (105) reported that strong seasonal presence of ARGs (Antibiotic Resistance Genes) within wastewater, with higher levels observed in autumn and winter which coincided with increased antibiotic prescribing in those months (105). Higher levels of resistance have been found in wastewater with higher antibiotic concentrations (e.g., hospitals discharge vs. municipality) (106). Understanding the relationship between antibiotic concentrations and resistance further could inform where to target mitigation measures more effectively.

### 3.3 Markers of pharmacological intervention

The proportion of regular pharmaceutical in wastewater has been assessed in numerous studies as a metric of disease prevalence. Analyses of metformin (a medication frequently used to treat type 2 diabetes), found in wastewater have been used to assess the prevalence of type 2 diabetes (107, 108). Measurement of pharmaceutical concentrations in wastewater has been used alongside non-wastewater indicators, such as survey data, socio-economic or demographic data, or environmental data to identify correlations (109).

Elevated levels of isoprostanes detected from wastewater, were suggested to be an indicator of increased levels of community anxiety during the COVID-19 (110). The use of these pharmaceutical biomarkers needs to be validated more, and extensive research is required to determine how the data may be used to improve public health measures.

## 4 Sample collection methods

### 4.1 Moore swab

The Moore swab was first proposed by Brendan Moore (111) to trace *S. paratyphi* B from sewage contaminated water in a small town in England (112, 113). In this method, a cotton gauze swab tied with string is submerged in water. The method traps pathogens as water passes through swab. After leaving it in water for 2–4 days, the swabs are sent to the laboratory inside sterile jars and processed further (111, 114). This method has been utilized throughout the world to detect several pathogens such as human norovirus, poliovirus, *E. coli*, *V. cholerae* and now SARS-CoV-2 as well.

**Figure 2:**
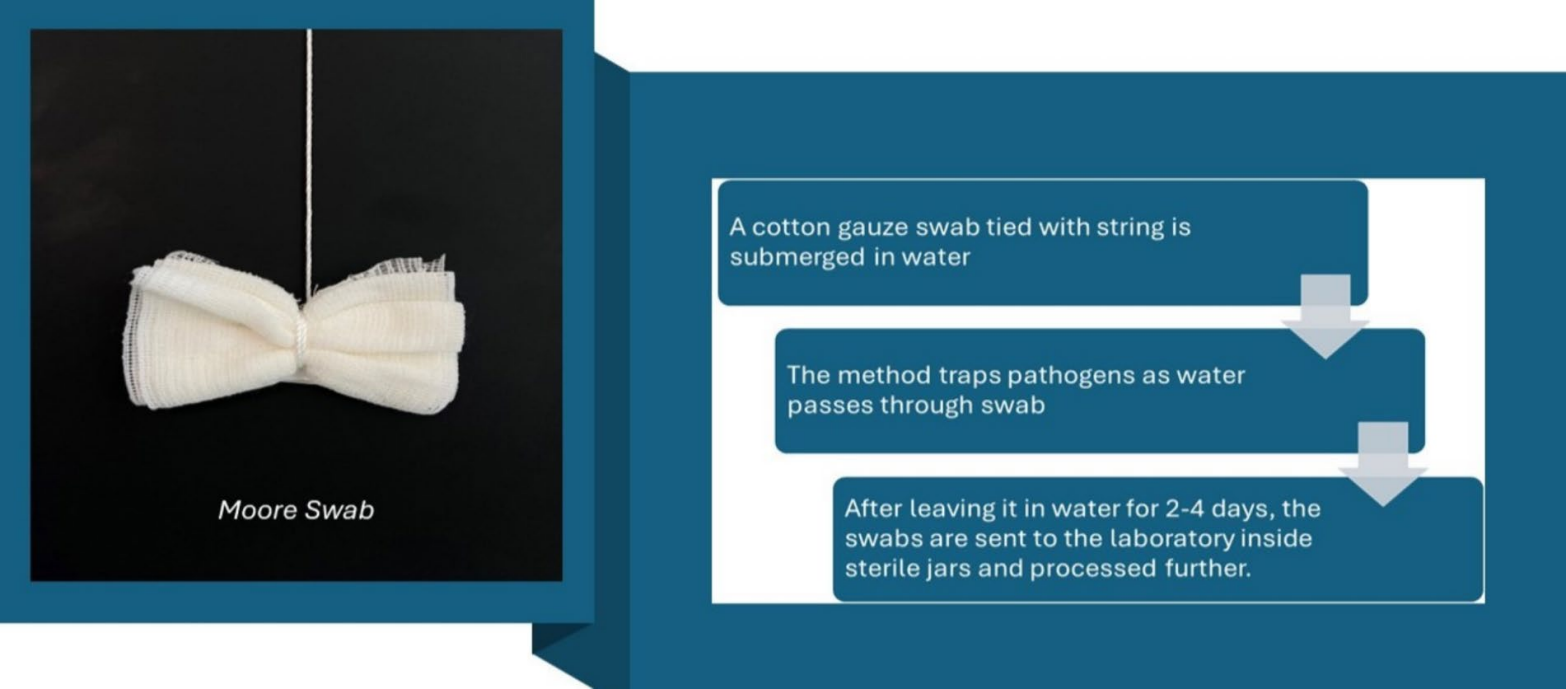
Moore swab collection method (a), (b) (111, 114, 121, 122).

Liu et al. (115), conducted a study in which Moore swab method was used for wastewater surveillance of COVID-19 at institutional level. Among the 219 swab samples tested, 28 (12.8%) swabs collected were found positive for SARS-CoV-2. Sbodio et al. (116), detected *E. coli* O157:H7 and *S. enterica* using Moore swab methodology in large volume field samples of irrigation water. Similarly, McEgan et al. (117), detected *Salmonella* spp. from larger volume of water by Moore swab method. In Farnham, United Kingdom, Hobbs (118) reported a case of typhoid in a 7-year-old child who had exposure to a sewage-contaminated river and the use of Moore swabs to trace the carrier. Greenberg et al. (119), and Shearer et al. (120), described detection of a single carrier in the isolated town of Portola, CA via use of Moore swabs in sewers; that carrier had been responsible for cases of typhoid occurring intermittently over 5 years (Figure 2).

### 4.2 Grab method

In this method, raw sewage is collected from sampling point either at 1 point in time or at specified points in time to form a composite sample. Many wastewater treatment plants use automated equipment to take samples at regular intervals during a 24-h period or during peak periods of domestic wastewater flow (122). The larger the volume of wastewater analyzed, higher the theoretical sensitivity to detect pathogen circulation in the source population (23). However, volumes greater than 1 L can be difficult to handle in the laboratory and can be replaced by multiple parallel regular samples.

**Figure 3:**
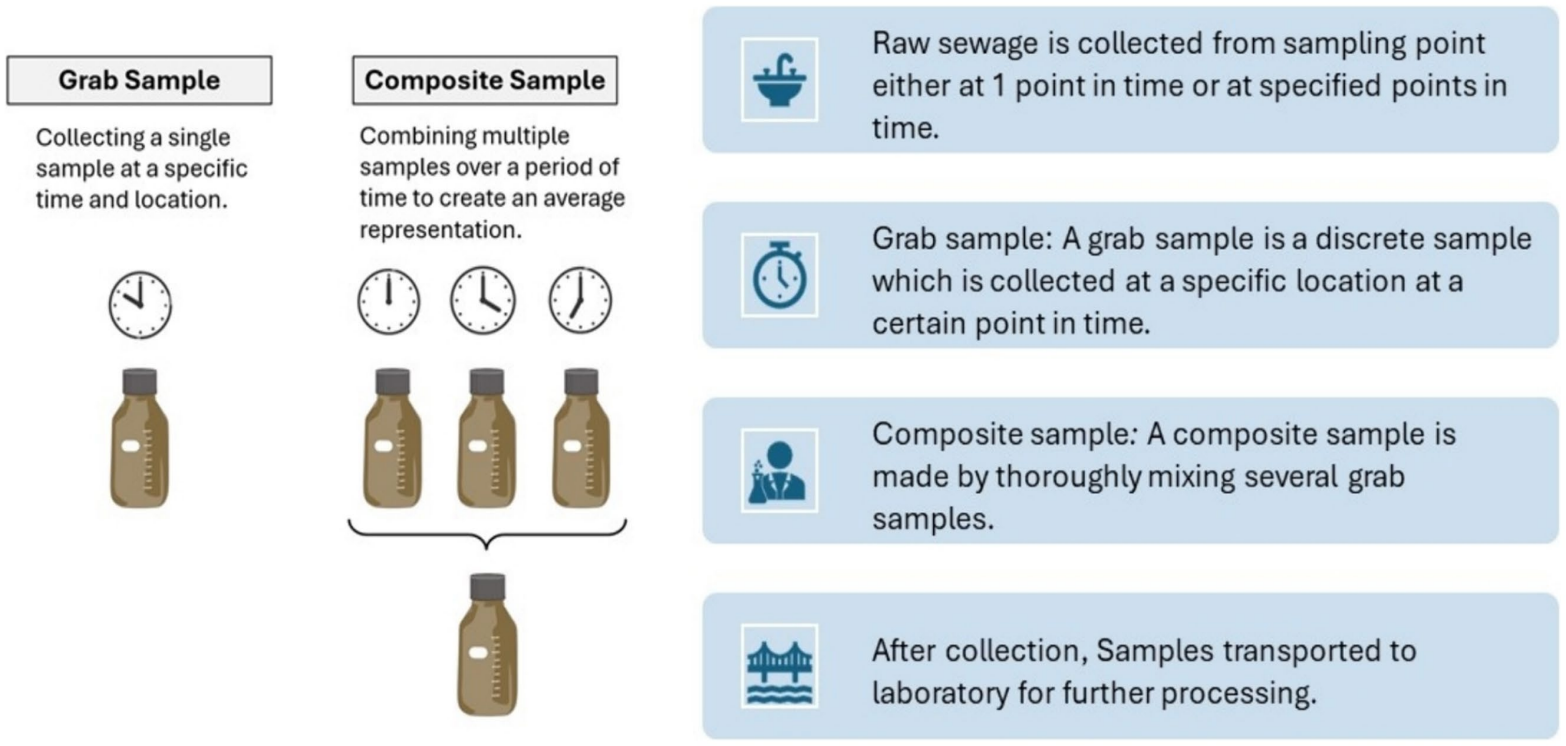
Grab and composite sampling methods (122–124).

Sampling is preferred to trapping because it is a more quantitative method that allows an estimation of the detection sensitivity of the system (123). In addition, long-term experience indicates that programs using concentrated sampling detect Polioviruses and non-polio enteroviruses more frequently than those using trap sampling (124) (Figure 3).

## 5 Methods available for detection of pathogens in wastewater

### 5.1 Culture based method

The utilization of culture-based approaches to capture antibiotic-resistant bacteria (ARB) is beneficial for various reasons such as verifying viability, testing for virulence (26), profiling phenotypic and genotypic multi-drug resistance (MDR) (125), and producing data that may be utilized for risk assessment related to human health. However, much of the media used to isolate opportunistic infections were not effective on environmental samples because they were created for clinical use.

Certain bacteria found in wastewater originate from the feces and can survive in surface water, while other populations of these bacteria are autochthonous and found in aquatic habitats. *Acinetobacter* spp., *Aeromonas* spp., and *Pseudomonas* spp., have been found to be important opportunistic pathogens that can grow in wastewater and natural aquatic environments. These pathogens can also acquire genes that confer multiple antibiotic resistance, making them potentially useful targets for culture-based monitoring (27).

The drawback of the culture-based approach is that, while some organisms may be inactivated (dead) or unable to grow on the chosen media (bacteria) or cell culture (used for viruses), molecular approaches can detect quantities from 1 to 10,000 greater than those of culture methods (126) (Table 2).

**Table 2:** Methods available for detection of pathogens in wastewater.

S.no.	Methods	Advantages	Disadvantages	Applications	References
1.	Culture based method	Verifies viability, tests for virulence, profiles MDR, produces data for risk assessment.	Not effective on environmental samples, slow, cannot detect inactivated or unculturable organisms.	Isolating ARB, opportunistic pathogens, and enteric bacteria.	Lagier et al. (26) and Joly-Guillou et al. (27)
2.	PCR	Fast, sensitive, specific, detects bacterial, viral, and protozoan pathogens.	Cannot discriminate viable from non-viable cells, low concentration of some pathogens, lacks data on infectious risk.	Detecting enteroviruses, HAV, *E. coli*, Cryptosporidium, Giardia, etc.	Law et al. (28) and Omar et al. (29)
3.	DNA microarray	Detects multiple targets in a single experiment, accurate, identifies low abundance species.	Expensive, complex probe design, affected by hybridization temperature, purity and degradation of genetic material, and amplification process.	Identifying 18 pathogenic bacteria, eukaryotes, and viruses; 941 pathogenic bacterial species in groundwater; 84 types of pathogens.	Severgini et al. (30) and Opitz et al. (31)
4.	FISH	Locates nucleic acids in cells or sample matrices, counts specific microbial populations, less sensitive to inhibitory substances	Can only detect a limited number of phylogenetically distinct targets simultaneously.	Detecting Salmonella spp., Enterobacteriaceae, *E. coli*, etc.	Santiago et al. (32) and Lukumbuzya et al. (33)
5.	LAMP	Isothermal, sensitive, specific, fast, detects pathogenic bacteria	Difficult to design specific primers.	Identifying Legionella spp., Leptospira spp., etc.	Niu et al. (34), Lu et al. (35), and Nzelu et al. (36)
6.	Pyrosequencing	Facilitates microbial genome sequencing, identifies bacterial species, strains, and mutations, analyzes genetic diversity of anti-microbial resistance.	Requires DNA templates at picomole level, expensive, complex, needs massive computing power.	Analyzing bacterial biofilm communities, potential pathogenic bacterial sequences, etc.	Wu et al. (37) and Peccia et al. (38)
7.	Digital PCR	Highly sensitive and robust, can detect multiplex viral targets Absolute Quantification.	Sample analysis cost and processing time typically higher than other PCR.	To detect and quantify SARS-CoV 2 variants, Greater precision and reproducibility in quantifying fecal markers.	Sedji et al. (39), Heijnen et al. (40), Cao et al. (41), and Tiwari et al. (42)
8.	Whole genome sequencing	Enables a comprehensive analysis of an individual’s entire genome Profiling of bacterial diversity and potential pathogens in wastewater.	Challenging due to low target concentration, complex microbial and chemical background, and lack of robust nucleic acid recovery experimental procedures.	Detect SARS-CoV-2, Norovirus GII, *E. coli* genotypes through RNA’s recovered from wastewater.	Behjati et al. (43), Crits-Christoph (44), and Fumian et al. (45)
9	MALDI-TOF MS	Rapid and accurate method of identification of bacterial and fungal isolates in the laboratory.	Relatively low resolving power compared to other high-resolution mass spectrometers.	Identification of *V. cholerae*, *V. alginolyticus*, *S. typhi*. Characterization of proteins present in wastewater.	Camacho et al. (46) and Rychert et al. (47)

### 5.2 Polymerase chain reaction

The identification of pathogens in wastewater can be accomplished by culture-based approaches, however the process can take many days or weeks. Without the requirement for cultivation, alternative molecular techniques like the PCR have proven successful in identifying bacterial, viral, and protozoan pathogens in sewage (127). PCR is the most common molecular-based technique to detect lesser amounts of a specific nucleic acid and is widely used for detection of pathogens (28). It enables the detection of a single pathogenic strain by targeting specific DNA sequences (28). This benefit makes it possible to identify and detect even lower amount of the target DNA sequence. It is thus widely used in the diagnosis of human pathogens (128). Fan et al. (129), reported PCR assay to achieve the simultaneous detection of various human pathogens in a single tube, with the detection sensitivities between 10 to 10^2^ CFU/100 mL in seawater. Omar et al. (29), identified commensal and pathogenic *E. coli* from medical and environmental water sources by using multiplex PCR technique. PCR technique, due to its high specificity, was also adopted to detection of enteroviruses and Hepatitis A virus (HAV) in environment.

Quantitative real-time PCR (qPCR), another PCR variant, allows for the measurement of DNA targets by tracking amplified products throughout cycle as evidenced by rising fluorescence (130). This approach decreases the potential of cross-contamination, offers excellent sensitivity and specificity, a faster rate of detection, and eliminates the requirement for post-PCR analysis (131). Shannon et al. (132), detected *E. coli*, *Klebsiella pneumoniae*, *Clostridium perfringens* and *Enterococcus faecalis* through wastewater by application of qPCR. With a lower quantification limit of 2.5 oocysts/sample, qPCR techniques have also been devised for the detection and identification of *Cryptosporidium* spp. in river water (133). qPCR had a sensitivity of 0.45 cysts per reaction for the detection of *G. lamblia* and *Giardia ardeae* in wastewater samples (134). For detection of RNA viruses, quantitative reverse-transcriptase (qRT)-PCR was developed to provide quantitative estimation of the pathogen concentration in water (135).

Limitations of PCR includes the inability to discriminate between viable from non-viable cells that both contain DNA, the low concentration of several pathogens in water such as *Cryptosporidium*, *Giardia* and viruses, and the lack of data to indicate the real infectious risk to a population (128, 131).

### 5.3 DNA microarray

One of the most innovative molecular biology-based techniques, DNA microarray technology enables researchers to run several environmental samples simultaneously in large-scale, data-intensive investigations (136). It is widely utilized to monitor gene expression under different cell growth conditions, detecting specific mutations in DNA sequences and characterizing microorganisms in environmental samples. It is a unique glass or silicon chip that has a DNA microarray that covers a surface area of several square centimeters with many nucleic acid probes. After being coupled with the probes, DNA, complementary DNA (cDNA), and RNA in the sample are identified by fluorescence or electric signal (137). DNA microarrays allow the hybridization-based detection of numerous targets in a single experiment. As a result, it is a quick and accurate diagnostic approach for analyzing several clinical or environmental samples (30). Wilson et al. (138), identified 18 pathogenic bacteria, eukaryotes, and viruses by using species-specific primer sets to amplify multiple regions unique toward individual pathogen in the microarray. Inoue and et al. (139) studied the occurrence of 941 pathogenic bacterial species in groundwater and were able to differentiate between human and animal sources. Leski et al. (140), developed a high-density re-sequencing microarray that has the capability of detecting 84 different types of pathogens ranging from bacteria, protozoa, and viruses, including *Bacillus anthracis*, Ebola virus and *Francisella tularensis* with detection limit of 104 to 106 copies per test for most of the pathogens exhibiting high specificity.

This technology is helpful as most known bacteria found in samples can be detected without the need for culturing, and the sensitivity of this approach allows for the detection of species with lower abundances (detection limit of 0.01% of microbial communities) (141). However, accuracy of the microarray data, complex probe design work, and clinical relevance of the early results have been criticized (127).

A single microarray experiment can be very expensive, there are many probe designs based on low-specificity sequences, and most widely used microarray platforms only use one set of manufacturer-designed probes, which leaves little control over the pool of transcripts that are analyzed. These are the main drawbacks of microarray technology. Along with their high sensitivity to changes in the hybridization temperature (142), the purity and rate of genetic material degradation (31), and the amplification process (143), microarrays also have other limitations. These factors, when combined, have the potential to affect gene expression estimates.

### 5.4 Fluorescent *in situ* hybridization

A cytogenetic method called FISH is used to locate the nucleic acids in cells or sample matrices. In molecular ecology, fluorescently labeled nucleic acid probes can be used to identify genes on chromosomes or to label ribosomal RNA in various taxonomic bacteria or archaea by hybridizing only with highly similar nucleic acids. It is possible to use FISH to count specific microbial populations (144).

Santiago et al. (32), detected Salmonella spp. from wastewater reused for irrigation by using FISH as a molecular method tool. Amann and Fuchs (144) isolated members of the family *Enterobacteriaceae* and *E. coli* in drinking water systems, freshwater and river water by this tool. In addition, emerging human pathogens in water, wastewater, sludge, and cellular survival and infection mechanisms have all been investigated with FISH (32, 33). Because it is less sensitive to inhibitory substances than PCR, FISH is better suited for complex matrices. However, the fact that only a limited number of phylogenetically distinct targets can be detected simultaneously is a major drawback of FISH.

### 5.5 Loop-mediated isothermal amplification

LAMP is a method for isothermal nucleic acid amplification. Currently, LAMP has been used to identify and quantify pathogenic bacteria with benefits in terms of sensitivity, specificity, and speed (145, 146). With a detection limit of 10 copies or less in the template for one reaction, the LAMP approach was also proven to be 10–100 times more sensitive than PCR detection (34). Lu et al. (35), utilized LAMP-based method for a rapid identification of Legionella spp. from the environmental water source. Koizumi et al. (147), used loop-mediated isothermal amplification method for rapid, simple, and sensitive detection of *Leptospira* spp. in urine sample.

This method can directly detect pathogenic microorganisms in wastewater avoiding the tedious step of culture and nucleic acid extraction (36). However, the major drawback of LAMP is it is more difficult to design specific primers for LAMP than for PCR (because LAMP requires 4–6 primers and PCR only two).

### 5.6 Pyrosequencing

Pyrosequencing is a DNA sequencing technique that facilitates microbial genome sequencing to identify bacterial species, discriminate pathogenic strains, and detect genetic mutations that confer resistance to anti-microbial agents (148). Hong et al. (149), analyzed bacterial biofilm communities in water meters of a drinking water distribution system by Pyrosequencing technique. Study conducted by Ibekwe et al. (150), identified most of the potential pathogenic bacterial sequences from three major phyla, namely, *Proteobacteria*, *Bacteroidetes*, and *Firmicutes* in a mixed urban watershed as revealed by pyrosequencing. The advantages of pyrosequencing for microbiology applications include rapid and reliable high-throughput screening and accurate identification of microbes and microbial genome mutations. The pyrosequencing instrument can also analyze the complete genetic diversity of anti-microbial drug resistance, including SNP typing, point mutations, insertions, and deletions, as well as quantification of multiple gene copies that may occur in some anti-microbial resistance patterns (151).

However, the DNA present in wastewater samples could limit the sensitivity of this tool as it requires DNA templates at picomole level, but a much lower amount of DNA can hamper the output (37, 38). This technology is also limited by the cost, the complexity of analysis, the need for increasing availability of massive computing power and the efficiency of data generation (152).

### 5.7 Digital PCR

To identify enteric virus contamination in water and wastewater, PCR and its variants such as quantitative PCR (qPCR), real-time RT-PCR, RT-qPCR, nested PCR, and digital PCR (dPCR) have been implemented (153). In contrast, qPCR can detect multiplex viral targets (154). Digital PCR (dPCR) has proven to be efficient for wastewater surveillance, owing to its increased robustness against PCR inhibitors commonly encountered in more difficult sample types (39, 155).

Heijnen et al. (40), evaluated that digital PCR may be utilized to detect and quantify mutations in SARS-CoV-2 in raw sewage samples from the cities of Amsterdam and Utrecht in The Netherlands. With its sensitivity and precision in quantification, digital PCR (dPCR) was quickly identified as a suitable choice for monitoring SARS-CoV-2 in wastewater monitoring (156). In terms of quantifying human-associated fecal markers in water, it was discovered that dPCR displayed superior precision and reproducibility than qPCR (41). With dPCR, the sample analysis cost and processing time are higher than qPCR. For the quantification of pathogens, dPCR can be a viable alternative if enhanced analytical performance (i.e., accuracy and sensitivity) is essential (42).

### 5.8 Whole genome sequencing

Profiling bacterial diversity and potential pathogens in wastewater has been a widely used application of sequencing, a robust analytical tool. For surveillance and outbreak investigations, the state of the art is shifting toward WGS (Whole Genome Sequencing) as a replacement for conventional molecular techniques (43, 157). WGS study of the complete pathogen genome has the potential to transform outbreak analysis by providing understanding of distinguishing even closely related bacterial lineages (158).

As demonstrated by Christoph et al. (44), numerous SARS-CoV-2 genotypes were found through sequencing of viral concentrations and RNA recovered directly from wastewater. Fumian et al. (45), identified Norovirus GII genotypes through genome sequencing from a wastewater treatment plant in Rio de Janeiro, Brazil. Mahfouz et al. (159), analyzed whole genome sequences for the indicator species *E. coli* of the inflow and outflow of a sewage treatment plant which revealed that nearly all isolates are multi-drug resistant, and many are potentially pathogenic. Recently, Mbanga et al. (160), reported genomics of antibiotic resistant *Klebsiella grimontii* novel sequence type ST350 isolated from a wastewater source in South Africa.

Whole genome sequencing reveals insights into recent improvements in sequencing technologies and analysis tools have rapidly increased the output and analysis speed as well as reduced the overall costs of WGS (158). Nevertheless, Genomic surveillance is still challenging due to low target concentration, complex microbial and chemical background, and lack of robust nucleic acid recovery experimental procedures (161).

### 5.9 MALDI-TOF

Matrix-assisted laser desorption ionization time of flight mass spectrometry (MALDI-TOF MS) is a rapid and accurate method of identification of bacterial and fungal isolates in the laboratory (162). The identification of microorganisms is based on the protein fingerprint unique to the microorganism (163, 164).

*V. cholerae* non-O1 isolates from wastewater were identified by MALDI TOF MS by Eddabra et al. (165). *V. alginolyticus* isolated from *Perna perna* mussles was efficiently identified by MALDI TOF MS by Bronzato et al. (166).

There are numerous studies that have proven the use of MALDI TOF MS on bacterial and fungal isolates. Croxatto et al. (167), have reported that numerous studies have been attempted to perform direct testing of urine using MALDI TOF MS. The method could be used with up to 94% accuracy but only if bacterial count is 105/ml. Nachtigall et al. (168), found that MALDI TOF was 80% concordant with RT-PCR in identifying SARS-CoV-2 from nasal mucus secretions. Rybicka et al. (169), found that MALDI TOF was better than RT-PCR in detecting SARS-Cov-2. Gerbersdorf et al. (170), have shown that dextran, gellan and xanthan from anaerobic microbial aggregates can be differentially demonstrated by MALDI TOF MS in different wastewater. The exopolysaccharides in biofilms are found to be important in microbial adhesion and aggregation (171). Picó et al. (172), found that MALDI TOF can be adapted for rapid detection and characterization of proteins in wastewater. However, MALDI-TOF MS has relatively low resolution power if compared to other high-resolution mass spectrometers and the accuracy of identification depends on the quality of the reference database (46, 47).

## 6 Challenges of wastewater-based epidemiology

### 6.1 Complexity of wastewater matrix

Although Wastewater-Based Epidemiology (WBE) offers appealing advantages for the monitoring of public health, it comes along with several challenges. One major challenge being the level of biomarkers (chemical and/or biological compounds) as it is far more diluted in wastewater which makes it difficult to trace (173). The complex matrix is also challenging for pathogen detection (174). Nucleic acid-based Polymerase chain reaction (PCR) is the primary technique for analyzing pathogens; however, wastewater contains a variety of PCR inhibitors, including fat, protein, and other compounds, that might affect PCR analysis (18).

### 6.2 Estimation of population size

The dynamic population size estimation is another challenge (175, 176). For example, it may be difficult to determine whether the presence of a pathogen in wastewater was caused by visitors passing through or by residents of the community in the concerned area (177). However, the presence of pathogens in wastewater, whether from the local population, undoubtedly provides valuable information, which may indicate an outbreak of disease in the community, thereby providing real time data for proper preparedness and response (178). This also ensures that WBE is used to provide timely warning of infectious disease outbreaks.

### 6.3 Detection methods

The physical distinctions between the major pathogen groups, the presence of inhibitors in the sample, established standard techniques for sample collection, culture-independent detection methods, and identification of pathogen host origin are the problems of detection methods (179). Specificity, sensitivity, repeatability of results, rapidity, automation, and cheap cost are the most significant prerequisites for reliable analysis (180). Furthermore, because human pathogens that reside in a viable but non-culturable (VBNC) form, such as *E. coli*, *Helicobacter pylori*, and *V. cholerae*, have a wide environmental dispersion, culture-dependent approaches may provide false negative results (28, 181).

## 7 Economics of wastewater surveillance

Performing clinical testing for mass surveillance puts a huge financial burden on low-and middle-income countries (LMICs), because WHO recommended testing protocols are costly to implement. In addition, the recent recommendation of the real-time surveillance of pathogens of concern that need prohibitively expensive next generation sequencing technology is less affordable by LMICs (182). While clinical surveillance will always be vital for the response to infectious diseases, wastewater-based surveillance allow for quick and economical surveillance–even in areas that are currently unexplored. Wastewater monitoring enables community prevalence quantification and rapid detection of pathogen. At sites where wastewater from the population collects and mixes, so do a diverse array of microbes shed from individuals (183). Pathogen concentrations accurately estimate prevalence (the number of current infections in the population) and given that wastewater trends often precede corresponding clinical detections, they may allow for early detection (184, 185).

To summarize, because wastewater surveillance covers a wide-scale population, the additional cost per resident would be very small, even when focusing on an institutionalized population. Primary screening with wastewater surveillance is highly likely to be economically more justifiable, scalable, providing results in real time than a primary screening with clinical tests. However, progressing toward more equitable and sustainable surveillance will require continued development of local, self-sustaining scientific ecosystems through laboratory and computational methods development and training, capacity building efforts, and financial support of domestic scientific enterprise.

## 8 Conclusion

Wastewater surveillance had shown great potential in providing complete health status information in a comprehensive and near-real-time manner at the community level. It offers a unique perspective on the spread and evolution of pathogens, aiding in the prevention and control of disease epidemics. This review underscores the importance of continued research and development in this field to overcome current challenges and maximize the potential of wastewater surveillance in public health. It also offers a framework and evidence foundation to guide laboratories in selecting the most suitable tools for implementing wastewater surveillance.

Since, there are so many emerging new pathogens that are causing illnesses and waterborne outbreaks, pathogen indicators need to be continually strengthened. Optimizing presently available technologies could increase our understanding of infectious pathogens, our ability to predict pathogen contamination, and our potential to safeguard public health. These technologies would be able to identify causal agents more precisely and quickly, detect viable microorganisms and characterize them according to microbial communities, and enable the creation of accessible data.

If wastewater monitoring is conducted consistently, it may be utilized to locate possible pathogen carriers, provide comprehensive data, determine the origin of the infections, and deliver reliable early warning. However, there is still a lot of work to be done for adoption on a broader scale.

## Author contributions

SS: Supervision, Visualization, Writing – original draft, Writing – review & editing. AmA: Writing – original draft. SuA: Writing – original draft. ShA: Writing – original draft. AsA: Writing – original draft. GO: Writing – original draft. MC: Writing – review & editing. SBS: Writing – original draft. GB: Writing – review & editing. WE: Writing – original draft, Writing – review & editing.
